# Describing the effect of COVID-19 on sexual and healthcare-seeking behaviours of men who have sex with men in three counties in Kenya: a cross-sectional study

**DOI:** 10.1136/sextrans-2024-056105

**Published:** 2024-07-04

**Authors:** Souradet Y Shaw, Jeffery C S Biegun, Stella Leung, Shajy Isac, Helgar K Musyoki, Mary Mugambi, Japheth Kioko, Janet Musimbi, Kennedy Olango, Samuel Kuria, Martin K Ongaro, Jeffrey Walimbwa, Faran Emmanuel, James Blanchard, Michael Pickles, Sharmistha Mishra, Marissa L Becker, Lisa Lazarus, Robert Lorway, Parinita Bhattacharjee

**Affiliations:** 1 Community Health Sciences, University of Manitoba, Winnipeg, Manitoba, Canada; 2 Faculty of Arts, University of Manitoba, Winnipeg, Manitoba, Canada; 3 India Health Action Trust, Delhi, India; 4 National AIDS and STI Control Programme, Ministry of Health, Nairobi, Kenya; 5 National Syndemic Disease Control Council, Nairobi, Kenya; 6 Partners for Health and Development in Africa, Nairobi, Kenya; 7 Men Against AIDS Youth Group, Kisumu, Kenya; 8 Mamboleo Peer Empowerment Group, Kiambu, Kenya; 9 HIV and AIDS People’s Alliance of Kenya, Mombasa, Kenya; 10 G10 Research Advisory Committee, Nairobi, Kenya; 11 University of Manitoba, Winnipeg, Manitoba, Canada; 12 Imperial College London, London, UK; 13 University of Toronto, Toronto, Ontario, Canada; 14 Institute for Global Public Health, University of Manitoba, Winnipeg, Manitoba, Canada

**Keywords:** HIV, COVID-19, Sexual and Gender Minorities

## Abstract

**Abstract:**

**Background:**

While the COVID-19 pandemic disrupted HIV preventative services in sub-Saharan Africa, little is known about the specific impacts the pandemic has had on men who have sex with men (MSM) in Kenya.

**Methods:**

Data were from an HIV self-testing intervention implemented in Kisumu, Mombasa and Kiambu counties in Kenya. Baseline data collection took place from May to July 2019, and endline in August–October 2020, coinciding with the lifting of some COVID-19 mitigation measures. Using endline data, this study characterised the impact the pandemic had on participants’ risk behaviours, experience of violence and behaviours related to HIV. Logistic regression was used to understand factors related to changes in risk behaviours and experiences of violence; adjusted AORs (AORs) and 95% CIs are reported.

**Results:**

Median age was 24 years (IQR: 21–27). Most respondents (93.9%) reported no change or a decrease in the number of sexual partners (median number of male sexual partners: 2, IQR: 2–4). Some participants reported an increase in alcohol (10%) and drug (16%) consumption, while 40% and 28% reported decreases in alcohol and drug consumption, respectively. Approximately 3% and 10% reported an increase in violence from intimate partners and police/authorities, respectively. Compared with those with primary education, those with post-secondary education were 60% less likely to report an increase in the number of male sexual partners per week (AOR: 0.4, 95% CI: 0.2 to 0.9), while those who were HIV positive were at twofold the odds of reporting an increase or sustained levels of violence from intimate partners (AOR: 2.0, 95% CI: 1.1 to 4.0).

**Conclusion:**

The results of this study demonstrate heterogeneity in participants’ access to preventative HIV and clinical care services in Kenya after the onset of the COVID-19 epidemic. These results indicate the importance of responding to specific needs of MSM and adapting programmes during times of crisis.

**What is already known on this topic**

- Few studies have documented the impact of COVID-19 on HIV-related risk and protective behaviours among men who have sex with men in Kenya, although evidence suggests heterogeneity in COVID-19’s impact on behaviours.

**What this study adds**

- The majority of participants reported minimal changes in alcohol and substance use, while behaviours and experiences of violence were linked to education level, place of residence and HIV status.

**How this study might affect research, practice or policy**

- Public health crises impact populations heterogeneously; understanding gaps in care as well as risk behaviours during times of crises can help tailor future interventions.

## Introduction

 Researchers noted the potential for the COVID-19 pandemic to reverse a decade’s worth of gains made in the response to the HIV epidemic in sub-Saharan Africa.^1 2^ Specifically, without adequate supplies of antiretroviral (ARV) therapy, coupled with interruptions to preventative modalities, such as condoms, peer education and HIV testing, increases in HIV mortality and incidence were expected.^1^ Fortunately, programmes were able to adapt^3 4^; for example, Kimani *et al* showed how programmes started distributing personal protective equipment, while also adapting how clinics were scheduled to meet with physical distancing guidelines.^3^ The distributions of both COVID-19 and HIV are shaped by overlapping inequities,^3 5^ such as socioeconomic marginalisation, and thus adverse impacts from both infections are shouldered by the most marginalised. Kenya has one of the largest HIV epidemics globally,^6^ with key populations, such as men who have sex with men (MSM), prioritised in national HIV responses.^7^,^10^ Same-sex sexual behaviours are criminalised in Kenya, leading to discrimination and limiting access to healthcare, increasing vulnerability of MSM to HIV and the direct and indirect impacts of COVID-19.^3 4 11 12^ Peer-driven approaches have been a critical feature of community-based responses to HIV in Nairobi; however, the government of Kenya did not officially recognise peer workers as essential workers during the first wave of COVID-19, and thus workers were not eligible to receive personal protective equipment and training during the pandemic.^4^

The first confirmed case of COVID-19 in Kenya was reported on 13 March 2020.^2^ As with other countries, the government responded with a series of non-pharmaceutical interventions including curfews, restrictions on movement, and closures of restaurants and bars.^3 13^ Some interventions were lifted at the end of June 2020. The pandemic disrupted health services delivery^14^ and had a deleterious impact on the socioeconomic security of Kenya’s most vulnerable populations.^4 15 16^ Although recent literature has detailed the adaptations MSM and MSM-specific HIV programmes made in response to COVID-19 in Kenya,^4 12^ little is known about the specific impacts the pandemic had on MSM, including programmatic-relevant changes in men’s sexual partnership patterns, and healthcare access and utilisation.^17^ Using data collected during the COVID-19 pandemic from the second round of serial cross-sectional integrated biological and behavioural assessments (IBBAs), implemented as part of the evaluation of a community-based HIV self-testing (HIVST) project,^18 19^ the main objective of our analyses was to characterise the pandemic’s impact on participants’ risk behaviours, experience of violence and behaviours related to the prevention and treatment of HIV infection, including utilisation of HIV-related services. As a secondary analysis, we characterised the places where men met their sexual partners. Finally, factors associated with behaviours and experience of violence were examined. Understanding specific vulnerabilities and gaps in care exacerbated by the COVID-19 pandemic can help inform population-specific responses during times of crises.^5 20^

## Methods

### Study setting

The University of Manitoba partnered with MSM-led community-based organisations (CBOs) and Kenya’s National AIDS & STI Control Programme (NASCOP) to design an intervention to promote HIVST in MSM communities in Kisumu, Mombasa and Kiambu counties.^18^ These sites were chosen because of high HIV prevalence, with self-reported prevalence among MSM between 13% and 23% in 2017,^7^ their large MSM communities^10^ and well-established community health infrastructure for MSM.^21^ The intervention targeted MSM above 15 years of age and used several delivery mechanisms for HIVST, including distribution through facility and community settings. As part of the evaluation of the HIVST project, serial cross-sectional IBBAs were conducted at baseline and endline, in May–July 2019 and August–October 2020, respectively; analyses are from the endline survey. We followed NASCOP’s guidelines in conducting sexual and reproductive health research with adolescent key populations whereby those 15 years and above are considered mature/emancipated minors.^22^ Since HIV testing in Kenya without a guardian is allowed for those 15 years and above,^23^ respondents were able to give consent and participate without guardian consent.

### Study design and participants

MSM were recruited from physical and virtual sites^18^; physical sites included locations such as bars, while virtual sites included web-based applications and social network sites. Endline data collection used the same sampling frame as the baseline survey; participants were included if they: (a) identified as male; (b) reported engaging in anal or oral sex with another male in the previous 12 months; and (c) were 15 years of age or above. A multistage cluster sampling approach involving physical and virtual sites was used to recruit 1200 participants (400 in each county); the methodology is described elsewhere.^18^ Briefly, a sampling frame was generated using programmatic mapping and size estimation of physical and virtual sites.^10 24 25^ Sites were sampled to recruit 200 MSM each from physical and virtual sites in each county. Recruitment involved random sampling of virtual and physical sites; for virtual sites, peer researchers used each selected site to further randomly recruit the predefined number of potential participants who were online when the peer researcher logged into the site. Respondents from both physical and virtual sites provided a list of known MSM contacts, from which a random sample of one contact was selected.

### Data collection

Data collection took place in private spaces (eg, CBOs), at a time and location chosen by participants. Individuals were then asked to visit the specified data collection site, where they were invited to provide written informed consent; participants were informed they could choose to participate in all or some elements of the IBBA. Trained researchers administered a face-to-face structured questionnaire in Kiswahili or English, at the participant’s choosing. Participants were offered HIV testing and counselling with a rapid two-test algorithm as per Kenya national guidelines,^26^ with on-site reporting of results. For positive results, participants were offered accompaniment to an MSM-focused clinic, or to a government testing and treatment clinic. All participants were provided with condoms, lubricants and information on HIVST. Those who were seronegative were offered HIV pre-exposure prophylaxis (PrEP). Participants were asked to provide a dried blood spot for HIV confirmatory serology, performed at the HIV National Laboratory in Nairobi, using the Bioelisa HIV test kit for screening and if positive, the Murex HIV1-2-O test for confirmation. Completed questionnaires were entered into an electronic database (CSPro, US Census Bureau and ICF International).^19^

### Measurement and data analysis

IBBAs were co-designed with community members, with the endline containing questions pertaining to the impact of COVID-19. These included questions on partnership patterns, service usage and experience of violence during the months between the onset of COVID-19 and June 2020. Three questions examined impact of COVID-19 on partnership patterns, including changes in the number of male sexual partners per week during the COVID-19 period, the average number of different male sexual partners participants had in a week and what locations they met their male sexual partners. Questions asked about service usage impact, including HIV testing, type of test accessed, whether men received PrEP and whether they received ARV. Finally, men were asked whether their alcohol use, drug consumption and experiences of violence from authorities and their intimate partners changed during the period of COVID-19 restrictions. The following binary outcomes were examined in bivariate and multivariable models: (1) increase in number of male sexual partners per week; (2) increase in alcohol use; (3) increase in substance use; (4) increased or sustained violence from police/authorities; and (5) increased or sustained violence from intimate partners. For the first three outcomes, responses were coded as ‘1’ if respondents answered ‘yes’ to whether number of male sexual partners, alcohol use and substance use had increased since the start of the COVID-19 pandemic in Kenya, respectively. Those answering ‘remained the same’ or ‘no’ were coded ‘0’. For the violence questions, those reporting ‘yes, there was an increase’ or ‘yes, remained the same’ were coded ‘1’, while those answering ‘no’ were coded ‘0’.

Sociodemographic characteristics at endline were described using proportions, means, medians and IQR, where appropriate. These characteristics include age group, county, highest level of education and monthly income; sexual behaviour including whether the participant preferred to meet their partners in physical sites, virtual sites or both,^18^ preferred sexual position/role, age at first anal/oral sex with a man, number of different male partners in the past 1 month, receipt of money or gifts in exchange for sex with a man (ever) and condom use at last sex with a male partner. The following variables were used in bivariate and multivariable logistic regression models: age group, county, highest level of education, places where male partners met, sexual position preference, received money/gift in exchange for sex with a man (ever), condom use with last male sexual partner and HIV status. Crude and adjusted ORs (AORs) and their 95% CIs are reported from logistic regression models. Data were analysed with SAS V.9.4 (SAS Institute) and visualised using R (V.4.2.2).

## Results

Of the 1239 participants included in the endline survey, 179 were excluded because they answered ‘don’t know’ or ‘N/A’ to at least one of the following questions, prefaced with ‘During the period of COVID-19, ‘was there a change in the number of male sexual partners you had per week?’; ‘On average, how many different male sexual partners did you have in a week?’; ‘Was there a change in your alcohol drinking behaviour in comparison with other months?’ or ‘Did your drug use behaviour change compared with other months?’. Respondents were also excluded if they had missing data on any of the explanatory variables used in this study. A total of 1031 participants were included in the final analytical sample, although some analyses included fewer participants, due to the type of question answered (eg, only those reporting drug use were retained for questions about changes in drug use). Based on the sample of 1031 participants (table 1), median age was 24 years (IQR: 21–27 years), with approximately one-third of participants (n=307, 29.8%) from Kiambu, while 388 (37.6%) and 336 (32.6%) participants were from Kisumu and Mombasa, respectively. Approximately 45% of participants had received post-secondary education. Most participants (68.4%) reported meeting partners at both physical and virtual sites. Approximately half of participants (48%) reported predominantly insertive sex, 62% reported ever receiving money or gifts in exchange for sex with a man, and 66% reported using condoms with their last male sexual partner. HIV prevalence was 19.6%.

**Table 1:** Selected characteristics, from endline survey, of men who have sex with men enrolled in HIV self-test study in Kisumu, Kiambu and Mombasa counties, Kenya, 2020 (N=1031)

Variable	Total
Age group	
Median (IQR)	24.0 (21.0–27.0)
<21	193 (18.7)
21–24	421 (40.8)
25–29	272 (26.4)
30+	145 (14.1)
County	
Kiambu	307 (29.8)
Kisumu	388 (37.6)
Mombasa	336 (32.6)
Highest level of education	
Up to primary	105 (10.2)
Secondary	464 (45.0)
Post-secondary	462 (44.8)
Places where male partners met	
Both physical and virtual sites	705 (68.4)
Physical sites	125 (12.1)
Virtual sites	201 (19.5)
Sexual position preference	
Predominantly receptive (bottom)	210 (20.4)
Predominantly insertive (top)	491 (47.6)
Both receptive and insertive	330 (32.0)
Received money/gift in exchange of sex with man (ever)	
No	395 (38.3)
Yes	636 (61.7)
Condom use with last male sexual partner	
No	348 (33.8)
Yes	683 (66.3)
HIV status	
Negative	829 (80.4)
Positive	202 (19.6)

Table 2 displays the impact COVID-19 had on participants. While some participants (6.1%) reported an increase in the period between the onset of the pandemic in Kenya and the endline survey, most experienced no change (50%) or a decrease (43.9%) in weekly number of male sexual partners. Of those reporting an increase in number of partners, the median number of unique partners was 2 (IQR: 2–4; online supplemental figure 1). Approximately 10% of participants reported an increase in alcohol, and of those reporting drug use, 16% reported an increase in use. Of those who answered (n=948), 11% (n=114) reported an increase in the amount of violence from police/authorities, while 2% (n=23) reported experiencing violence from this source, but at pre-pandemic levels. Of those participants who reported on their experience of violence from intimate partners (n=1029), 3% (n=34) reported an increase, while 4% (n=39) reported their experience of violence was unchanged from pre-pandemic times. Out of 185 eligible participants, 21.4% (n=40) reported being unable to obtain PrEP; similarly, of 84 eligible participants, only 1 reported being unable to obtain ARV. Out of 511 eligible participants, 20% were no longer able to obtain an HIVST; of 852 participants, 8.1% (n=69) could no longer meet with a peer educator and out of 828 participants, 4.1% (n=34) were unable to visit a drop-in clinic. Online supplemental figure 2 shows the types of HIV testing accessed for those due for an HIV test and were able to get tested. A total of 689 participants were due for an HIV test and received testing, while 201 participants (~20%) who were due for an HIV test reported that they could not get tested. The majority of respondents reported meeting their sexual partners through the internet and social media applications (online supplemental figure 3); of interest is the wide disparity in use of social media and other technologies between sites, with men in Kiambu more likely to report use of WhatsApp and Facebook than the other two sites. Approximately 70% of men in Kiambu reported using Facebook and WhatsApp to connect with male sexual partners; in Kisumu and Mombasa, 33% and 43% reported using Facebook, respectively.

**Table 2:** Change in self-reported risk behaviours, experiences of violence, and use of HIV and other clinical services of men who have sex with men at endline survey, HIV self-test (HIVST) study in Kenya, 2020

	Variable	N	Increased (N, %)	Remained the same (N, %)	Decreased (N, %)
Self-reported risk behaviours and experiences of violence	Change in number of male sexual partners per week	1031	65 (6.1)	530 (50.0)	465 (43.9)
	Was there a change in your alcohol behaviour?	1031	108 (10.2)	530 (50.0)	422 (39.8)
	Did your drug use behaviour change?	655	111 (16.4)	377 (55.6)	190 (28.0)
	Experience violence from police/authorities	948	114 (10.8)	23 (2.2)	838 (79.1)
	Experience violence from intimate partners	1029	34 (3.2)	39 (3.7)	985 (92.9)
			**Yes, same as before (N, %**)	**Yes, but more difficult (N, %**)	**No, could no longer get it (N, %**)
Use of HIV and other clinical services	Did you get PrEP?	185	131 (70.1)	16 (8.6)	40 (21.4)
	Did you get your ARV?	84	77 (91.7)	6 (7.1)	1 (1.2)
	Were you able to get HIVST?	511	333 (63.9)	84 (16.1)	104 (20.0)
	Were you able to meet a peer educator?	852	550 (64.6)	233 (27.3)	69 (8.1)
	Were you able to visit a drop-in clinic?	828	569 (68.7)	225 (27.2)	34 (4.1)

ARVantiretroviralPrEPpre-exposure prophylaxis

Table 3 shows the distribution of participants’ characteristics by the five outcome variables, while online supplemental table 1 shows results from bivariate analyses. In bivariate analyses and relative to those reporting only primary education, participants reporting secondary (OR: 0.5, 95% CI: 0.2 to 0.9) and post-secondary education (OR: 0.4, 95% CI: 0.2 to 0.7) were less likely to report an increase in the number of sexual partners per week. Relative to participants from Kisumu, participants from Kiambu were more likely to report increases in alcohol use (OR: 2.6, 95% CI: 1.6 to 4.3), as were those with post-secondary education (OR: 3.8, 95% CI: 1.3 to 10.6). In adjusted analyses (table 4) and at the p<0.05 level, post-secondary education remained significantly associated with being less likely to report an increase in male sexual partners (AOR: 0.4, 95% CI:0.2 to 0.9), relative to those with primary education. Participants with post-secondary education remained significantly more likely to report an increase in alcohol use (AOR: 3.4, 95% CI: 1.2 to 10.0). Finally, participants living with HIV were twofold more likely (AOR: 2.1, 95% CI: 1.1 to 4.0) to report increased/sustained violence from their intimate partners. In adjusted analyses, the association between virtual sites and increased/sustained violence (from police/authorities and intimate partners) was no longer statistically significant at the p<0.05 level.

**Table 3:** Selected characteristics of men who have sex with men enrolled in HIV self-test study in Kisumu, Kiambu and Mombasa counties, by changes in risk behaviours and experiences of violence (Kenya, 2020)

	Increase in male sexual partners per week (N=1031)		Increase in alcohol use (N=1031)		Increase in drug use (N=655)		Increased/sustained violence from police/authorities (N=948)		Increased/sustained violence from intimate partners (N=1029)	
	**Yes (N, %**)	**No (N, %**)	**Yes (N, %**)	**No (N, %**)	**Yes (N, %**)	**No (N, %**)	**Yes (N, %**)	**No (N, %**)	**Yes (N, %**)	**No (N, %**)
Age group										
<21	12 (18.8)	181 (18.7)	17 (15.7)	178 (18.7)	20 (18.4)	96 (17.6)	17 (12.8)	161 (19.8)	10 (13.7)	183 (19.1)
21–24	27 (42.2)	394 (40.4)	47 (43.5)	393 (41.3)	49 (45.0)	225 (41.2)	63 (47.4)	323 (39.6)	33 (45.2)	388 (40.6)
25–29	15 (23.4)	257 (26.6)	29 (26.9)	249 (26.2)	27 (24.8)	156 (28.6)	38 (28.6)	216 (26.5)	18 (24.7)	254 (26.6)
30+	10 (15.6)	135 (14.0)	15 (13.9)	131 (13.8)	13 (11.9)	69 (12.6)	15 (11.3)	115 (14.1)	12 (16.4)	131 (13.7)
County										
Kiambu	16 (25.0)	291 (30.1)	50 (46.3)	263 (27.6)	40 (36.7)	181 (33.2)	7 (5.3)	290 (35.6)	7 (9.6)	299 (31.3)
Kisumu	20 (31.3)	368 (38.1)	27 (25.0)	369 (38.8)	44 (40.4)	141 (25.8)	109 (82.0)	276 (33.9)	46 (63.0)	342 (35.8)
Mombasa	28 (43.8)	308 (31.9)	31 (28.7)	320 (33.6)	25 (22.9)	224 (41.0)	17 (12.8)	249 (30.6)	20 (27.4)	315 (33.0)
Highest level of education										
Up to primary	13 (20.3)	92 (9.5)	4 (3.7)	107 (11.3)	12 (11.0)	62 (11.4)	9 (6.8)	83 (10.2)	9 (12.3)	96 (10.0)
Secondary	29 (45.3)	435 (45.0)	44 (40.7)	425 (44.7)	46 (42.2)	252 (46.2)	63 (47.4)	363 (44.5)	31 (42.5)	433 (45.3)
Post-secondary	22 (34.4)	440 (45.5)	60 (55.6)	419 (44.1)	51 (46.8)	232 (42.5)	61 (45.9)	369 (45.3)	33 (45.2)	427 (44.7)
Places where male partners met*										
Both physical and virtual sites	45 (70.3)	660 (68.3)	68 (63.0)	637 (69.0)	68 (62.4)	368 (67.4)	107 (80.5)	543 (66.6)	60 (82.2)	643 (67.3)
Physical sites	7 (10.9)	118 (12.2)	12 (11.1)	113 (12.2)	14 (12.8)	64 (11.7)	18 (13.5)	86 (10.6)	7 (9.6)	118 (12.3)
Virtual sites	12 (18.8)	189 (19.5)	28 (25.9)	173 (18.7)	27 (24.8)	114 (20.9)	8 (6.0)	186 (22.8)	6 (8.2)	195 (20.4)
Sexual position preference										
Predominantly receptive	16 (25.0)	194 (20.1)	22 (20.4)	188 (20.4)	22 (20.2)	110 (20.2)	25 (18.8)	172 (21.1)	14 (19.2)	196 (20.5)
Predominantly insertive	25 (39.1)	466 (48.2)	48 (44.4)	443 (48.0)	54 (49.5)	255 (46.7)	76 (57.1)	377 (46.3)	37 (50.7)	454 (47.5)
Both receptive and insertive	23 (35.9)	307 (31.8)	38 (35.2)	292 (31.6)	33 (30.3)	181 (33.2)	32 (24.1)	266 (32.6)	22 (30.1)	306 (32.0)
Received money/gift in exchange of sex with man (ever)										
No	23 (35.9)	372 (38.5)	47 (43.5)	348 (37.7)	37 (34.0)	210 (38.5)	32 (24.1)	336 (41.2)	17 (23.3)	377 (39.4)
Yes	41 (64.1)	595 (61.5)	61 (56.5)	575 (62.3)	72 (66.1)	336 (61.5)	101 (75.9)	479 (58.8)	56 (76.7)	579 (60.6)
Condom use with last male sexual partner										
No	19 (29.7)	329 (34.0)	36 (33.3)	312 (33.8)	34 (31.2)	211 (38.6)	41 (30.8)	272 (33.4)	26 (35.6)	322 (33.7)
Yes	45 (70.3)	638 (66.0)	72 (66.7)	611 (66.2)	75 (68.8)	335 (61.4)	92 (69.2)	543 (66.6)	47 (64.4)	634 (66.3)
HIV status										
Negative	48 (75.0)	781 (80.8)	85 (78.7)	744 (80.6)	93 (85.3)	436 (79.9)	124 (93.2)	643 (78.9)	57 (78.1)	772 (80.8)
Positive	16 (25.0)	186 (19.2)	23 (21.3)	179 (19.4)	16 (14.7)	110 (20.2)	9 (6.8)	172 (21.1)	16 (21.9)	184 (19.3)

* Physical sites include hotspots, nightclubs, etc.; virtual sites include social media, dating applications, etc.

**Table 4:** Adjusted ORs (AORs) and 95% CIs from multivariable logistic regression models, association between selected characteristics and reported changes in risk behaviours and experiences of violence of men who have sex with men enrolled in HIV self-test study in Kisumu, Kiambu and Mombasa counties (Kenya, 2020)

	Increase in male sexual partners per week	P value	Increase in alcohol use	P value	Increase in drug use	P value	Increased/sustained violence from police/authorities	P value	Increased/sustained violence from intimate partners	P value
	AOR (95% CI)		AOR (95% CI)		AOR (95% CI)		AOR (95% CI)		AOR (95% CI)	
Age group										
<21	1.23 (0.47, 3.21)	0.676	0.70 (0.32, 1.53)	0.373	0.84 (0.37, 1.91)	0.676	0.54 (0.24, 1.21)	0.132	0.72 (0.28, 1.84)	0.490
21–24	1.34 (0.59, 3.04)	0.484	0.88 (0.46, 1.68)	0.700	0.91 (0.44, 1.86)	0.786	1.08 (0.56, 2.09)	0.814	0.97 (0.46, 2.05)	0.946
25–29	1.07 (0.45, 2.55)	0.874	0.82 (0.42, 1.62)	0.564	0.76 (0.36, 1.63)	0.486	1.36 (0.67, 2.72)	0.393	0.83 (0.37, 1.83)	0.636
30+	Ref		Ref		Ref		Ref		Ref	
County										
Kiambu	0.97 (0.46, 2.06)	0.933	2.58 (1.47, 4.53)	0.001	0.76 (0.42, 1.37)	0.368	0.09 (0.04, 0.20)	<0.0001	0.18 (0.07, 0.44)	0.000
Kisumu	Ref		Ref		Ref		Ref		Ref	
Mombasa	1.49 (0.78, 2.82)	0.227	1.50 (0.85, 2.65)	0.160	0.33 (0.19, 0.59)	0.000	0.18 (0.10, 0.32)	<0.0001	0.42 (0.23, 0.76)	0.005
Highest level of education										
Up to primary	Ref		Ref		Ref		Ref		Ref	
Secondary	0.50 (0.24, 1.05)	0.068	2.61 (0.89, 7.61)	0.080	0.77 (0.37, 1.62)	0.490	1.48 (0.67, 3.28)	0.332	0.81 (0.35, 1.85)	0.612
Post-secondary	0.39 (0.17, 0.86)	0.019	3.42 (1.17, 10.04)	0.025	0.89 (0.41, 1.92)	0.765	1.38 (0.62, 3.09)	0.434	0.93 (0.40, 2.13)	0.855
Places where male partners met										
Both physical and virtual sites	Ref		Ref		Ref		Ref		Ref	
Physical sites	0.73 (0.31, 1.72)	0.470	1.22 (0.61, 2.44)	0.565	1.48 (0.74, 2.95)	0.263	1.13 (0.62, 2.07)	0.684	0.66 (0.29, 1.53)	0.338
Virtual sites	0.98 (0.48, 2.00)	0.952	1.08 (0.65, 1.80)	0.773	1.59 (0.90, 2.81)	0.108	0.59 (0.27, 1.33)	0.205	0.58 (0.23, 1.45)	0.242
Sexual position preference										
Predominantly receptive	Ref		Ref		Ref		Ref		Ref	
Predominantly insertive	0.67 (0.34, 1.33)	0.251	0.93 (0.53, 1.65)	0.808	1.03 (0.58, 1.85)	0.915	1.05 (0.61, 1.79)	0.870	1.18 (0.60, 2.32)	0.633
Both receptive and insertive	0.86 (0.43, 1.70)	0.665	1.03 (0.58, 1.82)	0.933	0.91 (0.50, 1.68)	0.767	0.92 (0.50, 1.68)	0.775	1.06 (0.52, 2.17)	0.880
Received money/gift in exchange of sex with man (ever)										
No	Ref		Ref		Ref		Ref		Ref	
Yes	0.93 (0.53, 1.64)	0.795	1.05 (0.67, 1.63)	0.842	1.40 (0.86, 2.28)	0.175	1.40 (0.88, 2.24)	0.160	1.54 (0.86, 2.78)	0.150
Condom use with last male sexual partner										
No	Ref		Ref		Ref		Ref		Ref	
Yes	1.28 (0.73, 2.26)	0.386	1.12 (0.72, 1.73)	0.625	1.34 (0.85, 2.11)	0.210	0.87 (0.56, 1.34)	0.517	0.76 (0.45, 1.28)	0.301
HIV status										
Negative	Ref		Ref		Ref		Ref		Ref	
Positive	1.25 (0.64, 2.44)	0.518	0.87 (0.51, 1.49)	0.608	0.80 (0.42, 1.50)	0.476	0.56 (0.26, 1.19)	0.130	2.06 (1.06, 4.04)	0.034

## Discussion

The current study demonstrates some participants having increased difficulty accessing preventative HIV and clinical services in Kenya after the arrival of COVID-19. These results align with published literature on the impact of COVID-19. Rao *et al*, in a multicountry study of MSM, found that perceived access to HIV services was negatively impacted by country-level stringency of COVID-19 measures, suggesting that strategies to ensure continuity of care should be a priority for MSM in public health crises.^27^ The authors also found a substantial reduction in access to HIV providers by MSM living with HIV.^27^ Similarly, Muhula *et al*, using a sample of people living with HIV from Kibera settlement, demonstrated a substantial drop in HIV prevention, care and treatment services, including clients starting ARV during the first wave of COVID-19. The same study found an increase in PrEP dispensations,^2^ suggesting programmes were able to adapt to COVID-19 in Kenya. It is worth noting that the majority of participants in our study reported little change in their ability to access services during the first wave of the COVID-19 pandemic, suggesting programmes in the three study sites were also able to adapt to the needs of participants, aligning with a recent study from Nairobi.^12^

Our study showed that the pandemic resulted in some disruptions to HIV services, with 21.3% of eligible participants unable to access PrEP, while 8.5% of participants found it more difficult to access PrEP. Although 20% of eligible participants reported being unable to access a self-test, with another 16% reporting more difficulty accessing a self-test during this time, our group has demonstrated an overall increase in the use and awareness of HIVST between baseline and endline surveys,^28^ suggesting access was not homogeneous overall. We found a portion of participants who reported higher or similar levels of intimate partner or police violence during the first wave COVID-19. However, while previous findings have suggested marginalised and vulnerable populations, particularly women, were more likely to experience higher rates of violence during the beginning of the COVID-19 pandemic,^29^ the majority of participants in this study reported decreased experiences with violence. Intimate partner violence has been associated with a greater number of sexual partners in MSM populations,^30^ and therefore a decrease in intimate partner violence may be linked to a decreased number of partners during COVID-19. Additionally, many participants in this study reportedly met partners through the internet (online supplemental figure 3), which may explain some participants reporting decreased violence from police and/or authorities, as these ‘meet-ups’ were taking place predominantly online or at home. Further research should explore the context behind experiences of violence during the pandemic to inform tailored support for MSM.

### Strengths and limitations

Our study had a number of strengths, including sampling from both physical and virtual sites, biological data on HIV status and the availability of data at the start of the pandemic in Kenya. Our study also had a number of limitations. First, data were collected via face-to-face interviews, and thus subject to social desirability bias, which may have resulted in under-reporting of behaviours. Second, the sampling method included recruitment of a limited number of contacts and may have resulted in selection bias, introducing a higher degree of homogeneity in samples. It should be noted that we have used similar representative sampling techniques across different countries (eg, India, Pakistan, Nigeria and Kenya) and in different contexts (eg, MSM, female sex workers, people who inject drugs). Finally, information on the impact of COVID-19 on participants was collected only at the beginning of the pandemic in Kenya; thus, longer-term impacts are not captured in survey responses; however, we expect that the most acute effects of the pandemic were most likely felt nearer to the start of the pandemic in Kenya.

## Conclusion

In conclusion, it is apparent that services accessed by MSM in Kenya, as well as some risk and mitigation behaviours, were impacted by the COVID-19 pandemic. Although HIV programmes, led by community members, adapted to the direct and indirect impacts of the pandemic, heterogeneity in how MSM experienced those impacts exists, and points to the importance of population-specific responses during times of crises to meet protective and preventative healthcare needs.

## supplementary material

10.1136/sextrans-2024-056105 online supplemental file 1

## Data Availability

Data are available upon reasonable request.
