# Supplementary material for: Describing the effect of COVID-19 on sexual and healthcare-seeking behaviours of men who have sex with men in three counties in Kenya: a cross-sectional study
Source: Sex Transm Infect. 2024 Jul 4;100(8):e056105. doi: 10.1136/sextrans-2024-056105 (PMC11672033; doi:10.1136/sextrans-2024-056105)

Supplemental Table S1: Crude Odds Ratios (OR) and 95% Confidence Intervals (95% CI) from Bivariate Models, Association between Selected Characteristics and Reported Changes in Risk Behaviours and Experiences of Violence, Men who have Sex with Men (MSM) enrolled in HIV Self-Test Study in Kisumu, Kiambu, and Mombasa Counties (Kenya, 2020)

	Increase in Male Sexual Partners per Week<br>OR (95% CI)	p-value	Increase in Alcohol Use<br>OR (95% CI)	p-value	Increase in drug use<br>OR (95% CI)	p-value	Increase/Sustained violence from police/authorities<br>OR (95% CI)	p-value	Increase/Sustained violence from intimate partners<br>OR (95% CI)	p-value
**Age Group**										
<21	0.89 (0.37, 2.12)	0.796	0.83 (0.40, 1.73)	0.626	1.08 (0.51, 2.32)	0.837	0.75 (0.36, 1.55)	0.435	0.60 (0.25, 1.42)	0.241
21-24	0.92 (0.44, 1.95)	0.836	1.04 (0.57, 1.93)	0.890	1.13 (0.58, 2.20)	0.714	1.36 (0.75, 2.44)	0.308	0.89 (0.45, 1.78)	0.745
25-29	0.78 (0.34, 1.77)	0.547	1.02 (0.53, 1.97)	0.960	0.90 (0.44, 1.84)	0.764	1.31 (0.70, 2.43)	0.399	0.76 (0.36, 1.63)	0.482
30+	ref		ref		ref		ref		ref	
**County**										
Kiambu	1.08 (0.56, 2.10)	0.821	2.60 (1.59, 4.26)	0.000	0.72 (0.45, 1.16)	0.177	0.07 (0.03, 0.14)	<0.0001	0.18 (0.08, 0.39)	<0.0001
Kisumu	ref		ref		ref		ref		ref	
Mombasa	1.63 (0.90, 2.95)	0.106	1.32 (0.77, 2.27)	0.306	0.39 (0.23, 0.65)	0.000	0.16 (0.10, 0.28)	<0.0001	0.46 (0.27, 0.80)	0.006
**Highest Level of Education**										
Up to Primary	ref		ref		ref		ref		ref	
Secondary	0.46 (0.23, 0.90)	0.023	2.77 (0.97, 7.88)	0.056	0.91 (0.46, 1.78)	0.780	1.35 (0.69, 2.68)	0.384	0.80 (0.37, 1.74)	0.576
Post-Secondary	0.33 (0.17, 0.68)	0.002	3.83 (1.36, 10.77)	0.011	1.09 (0.56, 2.11)	0.807	1.30 (0.66, 2.58)	0.445	0.84 (0.39, 1.82)	0.661
**Places where Male Partners Met**										
Both physical and virtual sites	ref		ref		ref		ref		ref	
Physical sites	0.98 (0.45, 2.14)	0.968	0.98 (0.51, 1.86)	0.941	1.11 (0.59, 2.08)	0.745	0.98 (0.57, 1.70)	0.952	0.63 (0.28, 1.40)	0.253
Virtual sites	0.95 (0.49, 1.83)	0.880	1.55 (0.97, 2.48)	0.069	1.29 (0.79, 2.10)	0.317	0.21 (0.10, 0.45)	<0.0001	0.34 (0.14, 0.79)	0.013
**Sexual Position Preference**										
Predominantly receptive	ref		ref		ref		ref		ref	
Predominantly insertive	0.63 (0.33, 1.21)	0.162	0.90 (0.53, 1.52)	0.681	1.04 (0.61, 1.79)	0.889	1.42 (0.88, 2.30)	0.156	1.10 (0.58, 2.09)	0.763

Both receptive and insertive	0.93 (0.48, 1.80)	0.832	1.09 (0.62, 1.90)	0.766	0.89 (0.49, 1.60)	0.686	0.81 (0.47, 1.42)	0.461	0.99 (0.49, 1.97)	0.968
**Received money/gift in exchange of sex with man (ever)**										
No	ref		ref		ref		ref		ref	
Yes	1.07 (0.64, 1.81)	0.787	0.79 (0.53, 1.19)	0.259	1.27 (0.83, 1.95)	0.273	2.22 (1.46, 3.36)	0.000	2.16 (1.24, 3.77)	0.007
**Condom use with last male sexual partner**										
No	ref		ref		ref		ref		ref	
Yes	1.23 (0.71, 2.13)	0.466	1.00 (0.66, 1.53)	0.994	1.40 (0.90, 2.17)	0.134	1.14 (0.77, 1.69)	0.514	0.90 (0.55, 1.48)	0.681
**HIV status**										
Negative	ref		ref		ref		ref		ref	
Positive	1.42 (0.79, 2.55)	0.246	1.14 (0.70, 1.86)	0.603	0.68 (0.39, 1.21)	0.189	0.27 (0.13, 0.54)	0.000	1.19 (0.67, 2.12)	0.552

Supplemental Figure 1: Number of Different Male Sex Partners in the Past Week, by Perceived Change in Partnership Patterns, Men who have Sex with Men (MSM) enrolled in HIV Self-Test Study in Kenya During First Wave of COVID, 2020 (N=1,031)

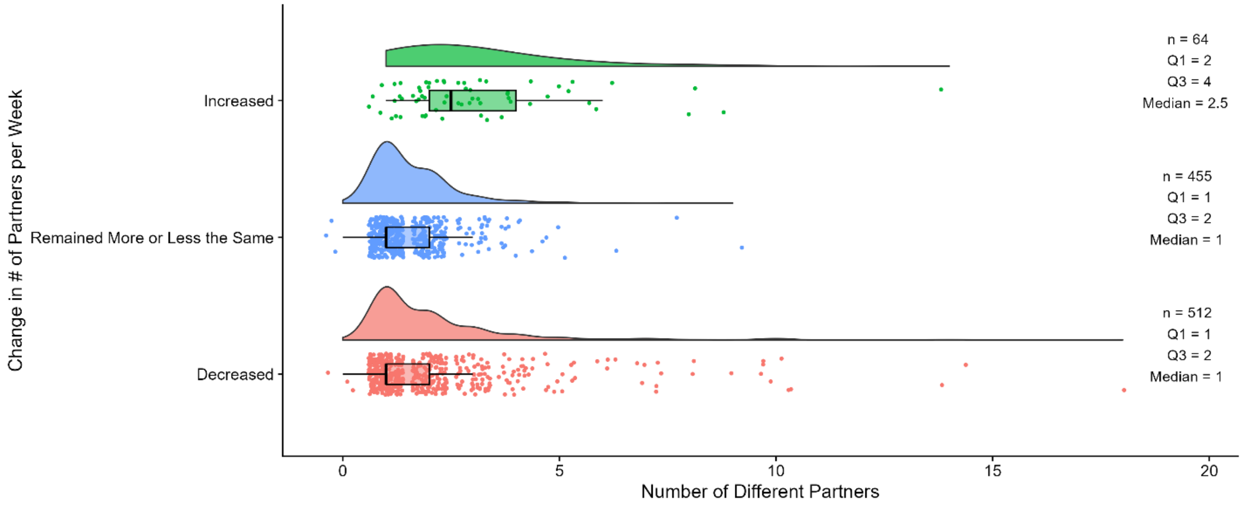

Supplemental Figure 2: HIV testing frequency and testing options used, Men who have Sex with Men (MSM) enrolled in HIV Self-Test Study in Kenya During First Wave of COVID, 2020 (N=1,031)

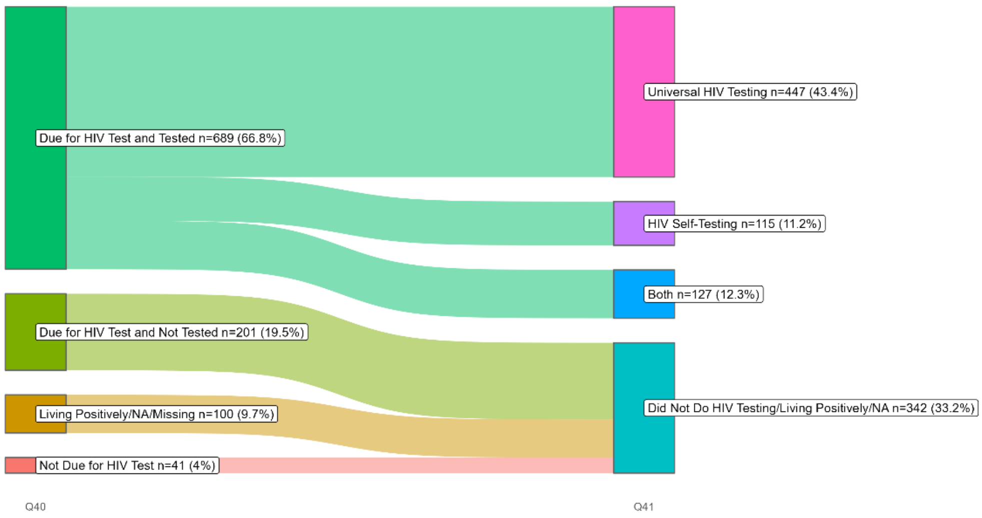

Supplemental Figure 3: Places where participants met sex partners, Men who have Sex with Men (MSM) enrolled in HIV Self-Test Study in Kenya During First Wave of COVID, 2020 (N=1,031)

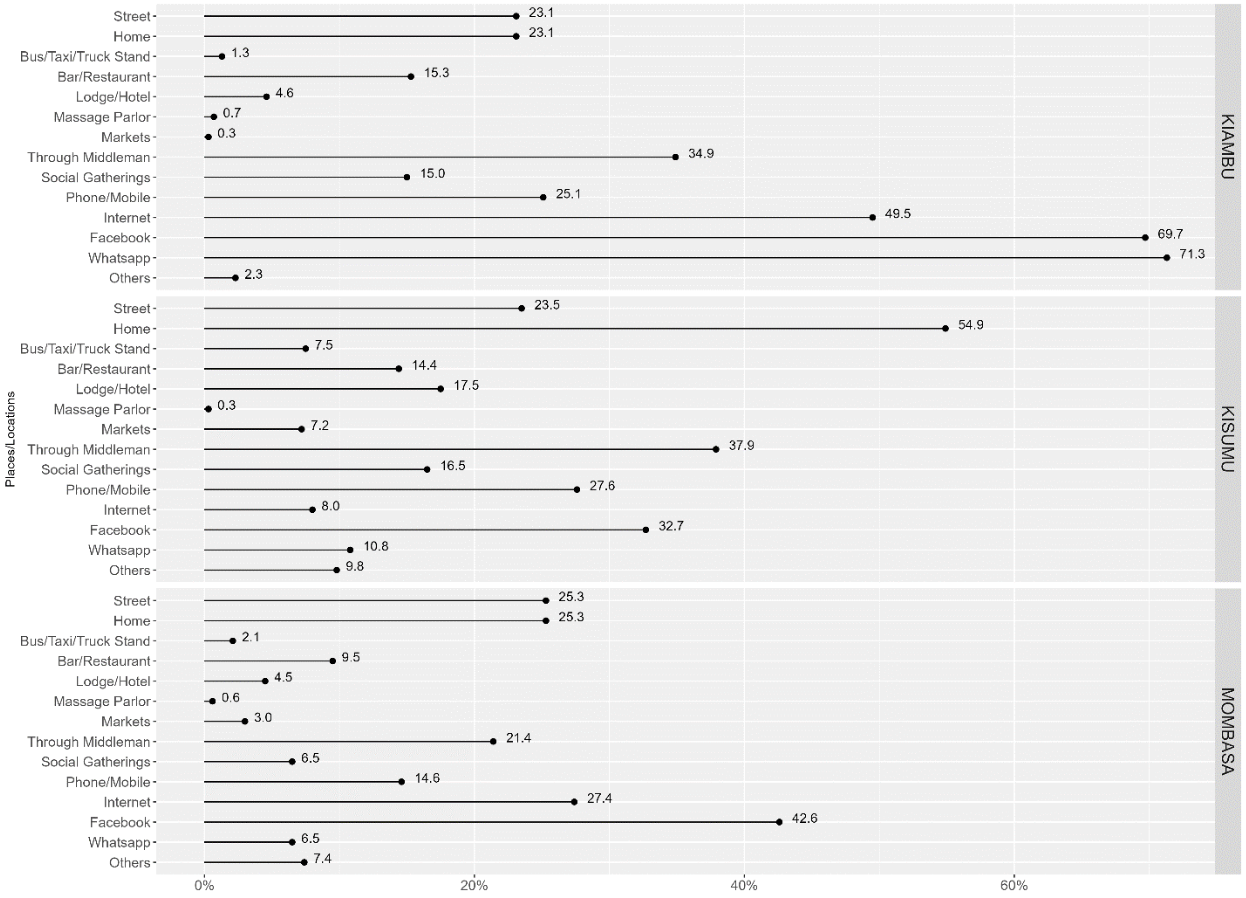

Supplement: online supplemental file 1 [file sextrans-100-8-s001.pdf]
